# The Relationship between the Conscious Self-Regulation of Schoolchildren’s Learning Activity, Their Test Anxiety Level, and the Final Exam Result in Mathematics

**DOI:** 10.3390/bs10010016

**Published:** 2019-12-26

**Authors:** Varvara Morosanova, Tatiana Fomina, Elena Filippova

**Affiliations:** Laboratory of Psychology of Self-Regulation, Psychological Institute of the Russian Academy of Education, Moscow 125009, Russia; morosanova@mail.ru (V.M.); proftest@gmail.com (E.F.)

**Keywords:** self-regulation, test anxiety, exam performance, learning activity

## Abstract

This article presents the results of a study on the relationship between conscious self-regulation of learning activity, test anxiety and performance in the Unified State Exam in mathematics in a sample of Russian students (N = 231). The Self-Regulation Profile of Learning Activity Questionnaire (SRPLAQ, 2015) and Spielberger’s Test Anxiety Inventory (TAI) (Russian adaptation, 2004) were used to measure self-regulation and anxiety, respectively. The study also took into account the students’ results for the Unified State Exam in mathematics. The study revealed a negative correlation for the test anxiety indicators with both the exam results and regulatory characteristics. The cluster analysis identified groups of students that differed in their level of self-regulation development, anxiety indicators, and the math exam result. It appears that students who have the lowest exam results are characterized not only by high test anxiety rates, but also by lower self-regulation levels. The regression analysis within the groups showed that a higher exam result is largely associated with a person’s regulatory resources. Examination success is based not so much on the ability to cope with adverse functional states, but on the maturity and stability of an integrated system of conscious self-regulation, which determines students’ effectiveness in achieving educational goals.

## 1. Introduction

Test anxiety has been studied for many years, but it still remains a serious educational problem. Scientists note that the test anxiety phenomenon will be relevant as long as test technologies are used for evaluating academic achievement [1,2].

In the research literature, test anxiety is considered as a combination of two components: worry and emotionality [3,4,5,6,7,8]. Worry is defined as a cognitive component, a comprehension of difficult-to-explain or disturbing circumstances, while emotionality is defined as non-specific reactions caused by stimulation of the autonomic nervous system. The emotional component is most pronounced in the period immediately preceding the testing procedure (or exam), and then, as a rule, decreases. The cognitive component is more stable over time and reaches greatest intensity during and after examinations. The analysis of the relationship between these two components and academic success suggests that the worry component influences exam results to a greater degree [3,9]. Worry has been found to be significantly negatively related to examination performance [4,9,10]. Currently, many researchers consider test anxiety as a multidimensional phenomenon, which, in addition to the described two components, also includes an interference component (i.e., taps distracting and blocking cognition that disturb or interrupt performance during exams) and a lack of confidence [11,12].

The meta-analysis data suggest that anxiety rates are negatively associated with academic achievement [13,14]. Researchers, however, emphasize that test anxiety does not adversely affect all types and forms of assessing students’ knowledge. Thus, while it has been shown that test anxiety is a significant negative predictor of overall grades and exam performance, it does not affect everyday classwork marks [15].

The increasing complexity of educational activities in the modern Russian school and the implementation of a system for the assessment of educational achievements based on testing with Unified State Examinations (USE) has increased the number of students who, while showing generally good knowledge of the subject, do not always successfully demonstrate this in the exam situation. Accordingly, there is social demand for the development of certain psychological competencies that allow students not only to learn successfully, but also to cope effectively with examinations.

Recent studies have shown that examination performance is highly influenced by the motivation to achieve, goal setting, self-esteem, level of aspiration, self-efficacy, self-confidence, engagement and self-concept [16,17,18,19,20,21,22]. The study of individual differences with regard to the factors that determine students’ anxiety is a topical research area. Some capable students may experience worry due to the unrealistic expectations of parents, peers, or teachers. These expectations may increase their anxiety in exam situations. For some less capable students, such expectations can also cause performance problems [2]. With regard to gender differences, most researchers report higher levels of test anxiety in females [16,23]. This is mainly attributed to their higher emotionality. At the same time, according to most researchers, academic success in girls is higher than in boys [24]. Zeidner (1990) found that when statistically controlling for academic aptitude, the influence of gender on test anxiety was minimal [25].

As the research data show, it is the mathematics exam that causes the most fear and difficulties for the majority of Russian students because it is designed in a way that makes it impossible to complete all the tasks using only the knowledge gained from studying the school curriculum and without special training. Thus, the question arises: what factors will help students with different levels of knowledge maintain an optimal level of anxiety to ensure a positive result in exams?

We suggest that acquiring the psychological competencies that allow students to successfully pass examinations is highly related to the development of the conscious self-regulation of educational activities, that is, the student’s ability to independently and responsibly promote educational and life goals and manage their achievement based on the maximum use of individual resources. Our research results suggest that conscious self-regulation is the most important psychological mechanism for the mobilization and actualization of an individual’s cognitive and personality resources [26]. We have demonstrated the influence of conscious self-regulation on academic success, and have investigated the role of self-regulation in the system of cognitive and intrapersonal predictors for achieving educational goals [27,28]. We have also proved its significant contribution to ensuring not only academic success, but also the reliability of students’ performance in exam situations, that is, an ability to maintain a sustainable result in the tense, psychologically difficult conditions of achieving educational goals [26]. Recent studies have revealed the mediator role of conscious self-regulation in the relationship between the test anxiety of schoolchildren and their exam results [27].

In our studies, conscious self-regulation is characterized as a cognitive-personal construct. It represents a system of cognitive mechanisms for information processing including planning, modeling, programming, and results evaluation. The individual peculiarity of these processes (their individual profile) at the personality level is represented by a number of instrumental personal-regulatory properties: flexibility, independence, reliability, etc. This structure emphasizes the meta-nature of conscious self-regulation as a psychological means of mobilizing and integrating both cognitive and personality resources for resolving various tasks of human activity [26].

Since conscious self-regulation includes deliberate processes that can be perceived (in principle), we have assumed that it will be primarily associated with the cognitive component of anxiety or worry. The level of worry, which increases during examination, leads to certain failures in the cognitive processes (for example, a lack of working memory, reduced attention or concentration, etc.), which should be compensated for by additional effort. In this sense, conscious self-regulation acts as a necessary tool for activating cognitive processes in the stressful conditions of an exam. So, in the present study we investigated the relationship between the indicators of conscious self-regulation, test anxiety and Unified State Exam success; identified student groups with different levels of the characteristics under study; and made a comparative analysis of the predictors of exam success among students with different levels of test anxiety.

## 2. Materials and Methods

### 2.1. Participants and Procedures

The study involved high school students in Grade 11 of Russian State schools in Moscow city aged from 16 to 18 years (M = 17.31, SD = 0.48), 53% males. The total number of participants in the sample was 231. A survey of students was conducted individually and/or in group form. At the end of the academic year, the Unified State Exam results were collected after the school testing as well as data on annual algebra achievements. Parental and school consent was obtained for all participants. Analyses were carried out on the depersonalized data. The study was conducted in accordance with the Helsinki Declaration. Ethical agreement and consent for access to schools was provided by the Psychological Institute of the Russian Academy of Education (approval number 2017/6-132). 

### 2.2. Measures

For the assessment of conscious self-regulation, the Self-Regulation Profile of Learning Activity Questionnaire (SRPLAQ) was used [29]. It includes 67 statements that describe typical situations that involve achieving educational goals, and it generates ten scales: planning (e.g., I often try to set a certain amount of time needed to complete the learning task); modeling (e.g., Unexpected changes in the timetable throw me off my stride); programming (e.g., When preparing for a test (exam), I usually think over the order of studying the material); results evaluation (e.g., Even when I’m tired, I tend to study until I’m satisfied with the result); flexibility (e.g., If I need to get prepared for a lesson, I can work even in an uncomfortable and unfamiliar situation); independence (e.g., I use every opportunity to make reports in class); reliability (e.g., I do not postpone preparing for the lessons even if I’m tired or feel sick); responsibility (e.g., I do not give up preparing for the lessons even if I have to choose between studying and spending time with my peers); social desirability (e.g., I always admit my mistakes); and the general level of self-regulation. SRPLAQ was previously validated in a sample of 14–18 years old students (N = 702). The validation study showed that coefficients of internal consistency of items for each scale ranged from 0.58 to 0.76, indicating an overall reasonable homogeneity of the items in each scale. The subscales were significantly correlated with each other (r = 0.22–0.66, *p* < 0.001). Each statement is rated on a 4-point scale (Yes, Probably Yes, Probably No, No). The responses are then reduced to only “Yes” and “No”, by counting “probably yes/probably no” as “yes/no”, respectively. The “yes” responses are then added up (items are reversed if necessary), so that high scores (a maximum of 9 for each scale) denote high self-regulation.

For measuring the students’ anxiety arising in situations that assess their knowledge, skills and competencies, the Russian adaptation of Spielberger’s “Test Anxiety Inventory” (TAI) was used [30]. The TAI is a psychometric scale for measuring individual differences in test anxiety as a situation-specific personality trait. It includes 20 statements. Respondents are asked to answer how often they experience anxiety symptoms before, during and after exams. The TAI technique allows for the diagnosis of three indicators: test anxiety (20) and its two main components—worry (8) and emotionality (8). The Cronbach’s internal reliability coefficient obtained in our study for the emotionality scale was 0.87, and 0.92 for the worry scale. 

We used the students’ annual marks in mathematics (algebra) and results of the Unified State Exam in mathematics as measures of mathematical achievement. Russian schools assess students’ performance using a 5-point system, with grade 5 indicating excellent performance, 4—good performance, 3—satisfactory performance, 2—bad performance (fail), and 1—very bad fail. Most students receive grades of 3–5 for the year, with grade 2 being extremely rare, and grade 1 being practically unused. Unified State Exam Grades (score on a 0–100 scale) for mathematics were obtained from school records.

We analyzed data using IBM SPSS Statistics, version 21: ANOVA, correlation analyses, regression analyses, k means cluster analysis.

## 3. Results

### 3.1. Descriptive Statistics and Gender Differences

The descriptive statistics for all variables are presented in Table 1. 

The obtained results show that girls have significantly higher rates of test anxiety compared to boys. This fact is consistent with earlier data, for example, [23]. At the same time, boys pass exams more successfully than girls, although there are no gender differences in the overall annual mark. There were no significant gender differences in self-regulation indicators except for the “reliability” parameter, which was significantly higher in boys. 

### 3.2. Correlations

During the initial stage of the study, we conducted an analysis of the correlation between the indicators of self-regulation, test anxiety, annual academic success and the Unified State Examination results in mathematics. As expected, we found significant positive correlations between most regulatory indicators and math success and negative correlations between math success and test anxiety (see Table 2).

These results confirm the results of our colleagues and our previous research: it is worry that negatively affects academic performance, while the influence of emotionality is negligible. In addition, worry is only associated (negatively) with the USE result. We found no correlation between indicators of success and emotionality as a component of anxiety.

Next, we analyzed the direct relationship between conscious self-regulation and test anxiety (see Table 3).

Almost all the regulatory components are negatively related to test anxiety. In other words, students with a high level of anxiety are characterized by lower indicators of the conscious self-regulation parameters. There are more significant connections between the self-regulation indicators and the worry component than with the emotional component of test anxiety.

### 3.3. Cluster Analysis

**Figure 1:**
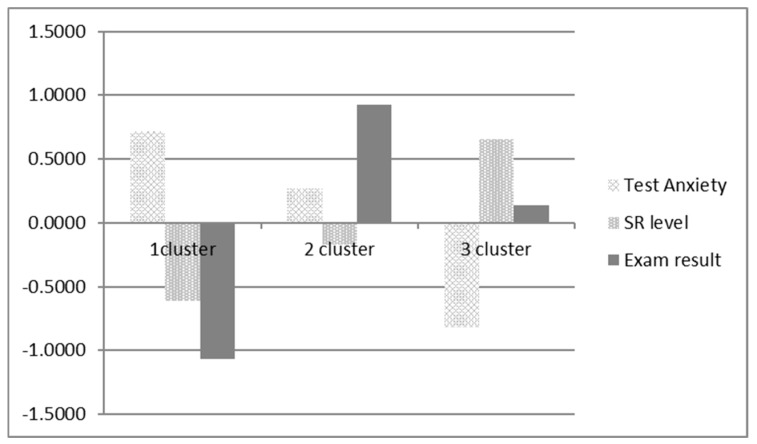
Clusters of students with different levels of anxiety, self-regulation, and USE result.

Cluster analysis was used to identify groups of students characterized by different levels of self-regulation, anxiety, and the USE result in mathematics. We used the k-means method, which allowed the identification of three typological groups of students (see Table 4 and Figure 1). Primary data have been transferred to z-scores.

The first group included students characterized, as compared to other groups, by the highest levels of test anxiety, the lowest levels of conscious self-regulation of learning activities and the lowest results for the Unified State Examination. The second cluster consisted of students with the highest USE results and medium levels of test anxiety and conscious self-regulation. The third cluster was made up of students with the lowest levels of anxiety, the highest levels of self-regulation and a fairly high USE result.

These results show that a sufficiently large group of children (32%) have difficulty regulating their learning activities and have a high level of test anxiety compared to their peers, and they also have the worse exam results (cluster 1). In our opinion, based on our previous studies, the development of conscious self-regulation is the basis for reliable academic performance [31], and in the case of high and medium test anxiety it can become a personal resource that helps to ensure the reliability of actions in stressful conditions, as can be seen in the students of clusters 2 and 3.

### 3.4. Regression Analyses

At the next stage of the analysis, we identified the regulatory predictors of exam success among the students in each cluster. For the students in clusters 1 and 3, anxiety (R2 = 0.16 and R2 = 0.07) turned out to be significant predictor with a negative effect; regulatory indicators were not identified as significant predictors. For the cluster 2 students (high USE results), significant predictors of exam success were regulatory indicators such as modeling (β = 0.311, *p* = 0.02), results evaluation (β = 0.388, *p* = 0.007), and reliability (β = 0.324, *p* = 0.009) with R2 = 0.33. This may indicate that in this group, self-regulation is a necessary mechanism to ensure an optimal exam result. Regression analysis showed that anxiety negatively affects exam results, while regulatory indicators are significant predictors in the group of students with the highest exam results. It can be assumed that practical work aimed at developing the conscious self-regulation of learning activities could effectively ensure more successful exam results in students with high levels of anxiety [32].

## 4. Discussion

In this study, we analyzed the specific relationships between conscious self-regulation of learning activities, test anxiety, and academic success in a sample of Russian schoolchildren. The students’ results for the Unified State Exam in mathematics served as a measure of academic success. As the research data show, it is the math exam that causes the most fears and difficulties for the majority of students because the testing form is designed in such a way that it seems impossible to complete all the tasks based on knowledge gained from studying the school program and without special training. Analysis of gender differences in samples from different countries have demonstrated that girls have significantly higher levels of test anxiety while boys have higher exam results [23], which suggests that boys’ academic performance is more reliable in stressful conditions. However, this discrepancy between genders appears to increase from primary grades to secondary grades, but then it decreases slightly when students are enrolled in post-secondary educational settings [14].

Our study results demonstrate that test anxiety is negatively associated with both academic grades and exam results, and that the exam result is more closely linked with worry and general levels of anxiety. This result is consistent with data from student samples in different countries [4,33,34]. Researchers report that students with medium levels of test anxiety pass exams more successfully. Students with high test anxiety as well as students with low test anxiety show lower academic performance [35]. 

According to meta-analysis data, the highest correlation between performance and test anxiety was identified among secondary school students who had taken standardized state exams [14].

Recent studies show that test anxiety, which badly influences performance, is also negatively related to self-regulation. The results obtained in this study provide empirical support in favor of the suggestion that an important condition for the successful passing of examinations is the ability of students to optimally regulate their learning activity. Conscious self-regulation allows the optimization of cognitive resources for achieving educational goals, and it is also a factor in reducing the impact of test anxiety on exam results.

According to researchers, 54% of Russian school graduates do not have sufficient math knowledge to successfully continue their education in fields that require the use of mathematics [36]. An analysis of Unified State Exam performance shows that in solving the examination tasks, students are very reliant on the use of algorithms without taking into account the specific conditions of a certain task. Also, students make a lot of computational mistakes. Students lack of success is often attributed to their inability to deviate from stereotyped formulations when they face unusual tasks, the so-called “stupor” state, when a graduate is unable to analyze a condition, etc. Self-regulation and self-efficacy can be factors that mediate the influence of anxiety on students’ academic achievements. In fact, many of the reasons for the low USE results in mathematics are connected with the inability to carefully analyze the problem, to think independently, go beyond the standard wording, with the ability to mobilize one’s cognitive resources, etc. It seems to us that the problems noted could be compensated for by the means and resources of conscious self-regulation.

For example, in our study of regulatory and cognitive predictors of different types of mathematical success, it was shown that the regulatory process, “modeling significant conditions” enhances the positive impact of some cognitive features and mathematical abilities on performance [27]. The level of “modeling” development allows students to detect and effectively use the conditions that are necessary for achieving goals. Our research demonstrates that conscious self-regulation not only directly influences exam results, but also acts as a mediator of the impact of students’ anxiety on their exam results. The higher the level of conscious self-regulation development, the lower the anxiety level and the better the exam results [37]. In this case, it seems natural that conscious self-regulation is primarily linked to the cognitive component of anxiety—worry. This conclusion is also based on a number of studies showing that it is the cognitive manifestations of anxiety that distract a person from solving a task, while emotionality plays an insignificant role in reducing exam results [1,38]. The questionnaire that we developed, the Self-Regulation Profile of Learning Activity Questionnaire (SRPLAQ) consists of scales for measuring the level of development of the basic cognitive processes: planning, modeling significant conditions for goal achievement, programming, and evaluating results. From our point of view, the basis for successfully passing examinations is not just the ability to cope with adverse functional states, but a well-developed and stable system of conscious self-regulation, which provides the tools for generating and achieving goals. In this sense, conscious self-regulation could be an important mechanism for reducing anxiety both at the stage of preparation for exams and at the stage of performance and reflective assessment. Programs designed to reduce test anxiety through the development of conscious self-regulation have proven their effectiveness in a number of Russian schools. 

## 5. Conclusions

The study identifies and describes groups of students with different levels of conscious self-regulation, test anxiety, and exam results. It was demonstrated that high test anxiety negatively affects exam results. The regulatory indicators proved to be significant predictors of mathematical success in the group of students with high exam results. As for the study limitations, it should be noted that it did not take into account some potentially important aspects of exam success, for example, self-concept and school engagement. In future research, the cross-cultural features of test anxiety in Russian students compared to students of other countries should also be considered. 

## Figures and Tables

**Table 1:** Means, standard deviations and ANOVA results by gender.

	Measures	ALL (N = 231)		Females (N = 108)		Males (N = 123)		ANOVA Effects
		M	SD	M	SD	M	SD	
Test Anxiety	Worry	18.81	7.31	20.37	7.46	17.43	6.92	0.002
	Emotionality	16.61	6.17	17.99	6.30	15.41	5.81	0.001
	Test Anxiety	41.14	11.40	44.02	11.53	38.62	10.71	0.000
Self-regulation	Planning	4.77	2.17	4.78	2.17	4.76	2.18	0.96
	Modelling	5.36	2.02	5.31	2.10	5.41	1.96	0.68
	Programming	5.16	1.83	5.07	1.78	5.24	1.88	0.48
	Result Evaluation	4.96	2.01	4.94	2.02	4.97	2.02	0.93
	Flexibility	5.75	2.13	5.67	2.23	5.82	2.05	0.58
	Independence	4.81	1.96	4.89	2.07	4.73	1.87	0.54
	Reliability	4.26	1.94	3.84	1.84	4.63	1.96	0.002
	Responsibility	4.12	2.28	4.11	2.21	4.13	2.35	0.95
	General Level of SR	39.16	10.84	38.69	10.43	39.59	11.21	0.53
Math	USE result in math	55.60	14.30	53.19	14.14	57.71	14.16	0.01
	Annual mark in algebra	3.83	0.69	3.79	0.67	3.87	0.712	0.36

**Table 2:** The correlation coefficients for the indicators of self-regulation, test anxiety, and mathematic achievement (Unified State Examination (USE) result and the annual mark in algebra) (N = 231).

Indicators	USE Result in Math	Annual Mark in Algebra
Planning	0.192 **	0.265 **
Modelling	0.225 **	0.255 **
Programming	0.107 **	0.210 **
Result Evaluation	0.229 **	0.197 **
Flexibility	0.221 **	0.150 *
Independence	0.078	0.062
Reliability	0.189 **	−0.009
Responsibility	0.174 **	0.213 **
General Level of SR	0.256 **	0.248 **
Worry	−0.461 **	−0.084
Emotionality	−0.084	−0.089
Test Anxiety	−0.267 **	−0.146 *

Note: * *p* < 0.05, ** *p* < 0.01.

**Table 3:** The correlation coefficients for the indicators of self-regulation and test anxiety (N = 231).

Indicators	Worry	Emotionality	Test Anxiety
Planning	−0.164 *	−0.141 *	−0.204 **
Modelling	−0.128	−0.205 **	−0.225 **
Programming	−0.155 *	−0.100	−0.187 **
Result evaluation	−0.167 *	−0.201 **	−0.271 **
Flexibility	−0.219 **	0.279 **	−0.321 **
Independence	−0.174 **	0.125	−0.180 **
Reliability	−0.330 **	0.312 **	−0.343 **
Responsibility	−0.132 *	−0.084	−0.158 *
General level of SR	−0.270 **	−0.265 **	−0.348 **

Note: * *p* < 0.05, ** *p* < 0.01.

**Table 4:** Mean values of anxiety, self-regulation, and USE result in the distinguished clusters (standardized scores).

Cluster	N	Test Anxiety	SR Level	Exam Result
1	73	0.7141	−0.6153	−1.0639
2	71	0.2665	−0.1724	0.9234
3	86	−0.8167	0.6570	0.1391

## References

[1] Tryon G.S. (1980). The measurement and treatment of test anxiety. Rev. Educ. Res..

[2] Wigfield A., Eccles J.S. (1989). Test anxiety in elementary and secondary school students. Educ. Psychol..

[3] Liebert R.M., Morris L.W. (1967). Cognitive and emotional components of test anxiety: A distinction and some initial data. Psychol. Rep..

[4] Cassady J.C., Johnson R.E. (2002). Cognitive test anxiety and academic performance. Contemp. Educ. Psychol..

[5] Morris L.W., Davis M.A., Hutchings C.H. (1981). Cognitive and emotional components of anxiety: Literature review and a revised worry–emotionality scale. J. Educ. Psychol..

[6] Schwarzer R. (1984). Worry and emotionality as separate components in test anxiety. Appl. Psychol..

[7] Spielberger C.D., Vagg P.R. (1995). Test Anxiety: A Transactional Process Model.

[8] Zeidner M. (1998). Test Anxiety: The State of the Art.

[9] Deffenbacher J.L. (1977). Relationship of worry and emotionality to performance on the Miller Analogies Test. J. Educ. Psychol..

[10] Morris L.W., Liebert R.M. (1970). Relationship of cognitive and emotional components of test anxiety to physiological arousal and academic performance. J. Consult. Clin. Psychol..

[11] Hodapp V., Benson J. (1997). The multidimensionality of test anxiety: A test of different models. Anxiety Stress Coping.

[12] Stöber J. (2004). Dimensions of test anxiety: Relations to ways of coping with pre-exam anxiety and uncertainty. Anxiety Stress Coping.

[13] Seipp B. (1991). Anxiety and academic performance: A meta-analysis of findings. Anxiety Res..

[14] Von der Embse N., Jester D., Roy D., Post J. (2018). Test anxiety effects, predictors, and correlates: A 30-year meta-analytic review. J. Affect. Disord..

[15] Pintrich P.R., De Groot E.V. (1990). Motivational and self-regulated learning components of classroom academic performance. J. Educ. Psychol..

[16] Bandalos D.L., Yates K., Thorndike-Christ T. (1995). Effects of math self-concept, perceived self-efficacy, and attributions for failure and success on test anxiety. J. Educ. Psychol..

[17] Elliot A.J., McGregor H.A., Gable S. (1999). Achievement goals, study strategies, and exam performance: A mediational analysis. J. Educ. Psychol..

[18] Lane J., Lane A.M., Kyprianou A. (2004). Self-efficacy, self-esteem and their impact on academic performance. Soc. Behav. Personal. Int. J..

[19] Vrugt A.J., Langereis M.P., Hoogstraten J. (1997). Academic self-efficacy and malleability of relevant capabilities as predictors of exam performance. J. Exp. Educ..

[20] Zimmerman B.J., Bandura A., Martinez-Pons M. (1992). Self-motivation for academic attainment: The role of self-efficacy beliefs and personal goal setting. Am. Educ. Res. J..

[21] Veiga F.H., García F., Reeve J., Wentzel K., García O. (2015). When adolescents with high self-concept lose their engagement in school. Rev. Psicodidáct..

[22] Garcia F., Martínez I., Balluerka N., Cruise E., García O.F., Serra E. (2018). Validation of the Five-Factor Self-Concept Questionnaire AF5 in Brazil: Testing factor structure and measurement invariance across language (Brazilian and Spanish), gender and age. Front. Psychol..

[23] Putwain D., Daly A.L. (2014). Test anxiety prevalence and gender differences in a sample of English secondary school students. Educ. Stud..

[24] Musitu-Ferrer D., Esteban-Ibañez M., León-Moreno C., García O.F. (2019). Is School Adjustment Related to Environmental Empathy and Connectedness to Nature?. Psychosoc. Interv..

[25] Zeidner M. (1990). Does test anxiety bias scholastic aptitude test performance by gender and sociocultural group?. J. Personal. Assess..

[26] Morosanova V.I. (2013). Self-regulation and personality. Procedia Soc. Behav. Sci..

[27] Morosanova V.I., Fomina T.G., Kovas Y., Bogdanova O.Y. (2016). Cognitive and regulatory characteristics and mathematical performance in high school students. Personal. Individ. Differ..

[28] Morosanova V.I., Fomina T., Bondarenko I.N. (2015). Academic achievement: Intelligence, regulatory, and cognitive predictors. Psychol. Russ..

[29] Morosanova V.I., Bondarenko I.N. (2015). Diagnostika samoregulyatsii cheloveka [Diagnosis of the Human’s self-Regulation].

[30] Karandashev V.N., Lebedeva M.S., Spilberger C. (2004). Izuchenie Otsenochnoy Trevozhnosti: Rukovodstvo po Ispol’zovaniyu Metodiki Ch.Spilbergera [Evaluation Study of Anxiety: A Guide to the Use of Ch.Spilberger’s Techniques].

[31] Morosanova V.I., Filippova E.V. (2019). Ot chego zavisit nadezhnost’ deistvii uchashchikhsya na ekzamene [What ensures reliability of a student’s actions in an examination]. Vopr. Psikhol..

[32] Filippova E.V., Fomina T.G., Morosanova V.I. (2015). Programma razvitiya osoznannoj samoregulyacii kak sredstva povysheniya nadezhnosti uchebnyh dejstvij uchashihsya v situacii ekzamena i eksperimentalnaya proverka ee effektivnosti [The program of development a conscious self-regulation as a means of improving the reliability of learning activities of students in the exam situation: Experimental verification of its effectiveness]. Vestn. Mosk. Univ. Ser. 14 Psikhol. [Mosc. Univ. Psychol. Bull.].

[33] Chapell M.S., Blanding Z.B., Silverstein M.E., Takahashi M., Newman B., Gubi A., McCann N. (2005). Test anxiety and academic performance in undergraduate and graduate students. J. Educ. Psychol..

[34] Rana R., Mahmood N. (2010). The relationship between test anxiety and academic achievement. Bull. Educ. Res..

[35] Putwain D.W., Daly A.L. (2013). Do clusters of test anxiety and academic buoyancy differentially predict academic performance?. Learn. Individ. Differ..

[36] Bolotov V.A., Sedova E.A., Kovaleva G.S. (2012). Sostoyanie matematicheskogo obrazovaniya v RF: Obshee srednee obrazovanie [The State of Mathematical Education in the Russian Federation: General Secondary Education (Analytical Review)]. Probl. Sovrem. Obraz. [Probl. Mod. Educ.].

[37] Morosanova V.I., Fomina T.G. (2017). Self-regulation as a Mediator in the Relationship between Anxiety and Academic Examination Performance. Procedia Soc. Behav. Sci..

[38] Sarason I.G. (1984). Stress, anxiety, and cognitive interference: Reactions to tests. J. Personal. Soc. Psychol..

